# Household transmission of the Delta COVID-19 variant in Queensland, Australia: a case series

**DOI:** 10.1017/S0950268822001546

**Published:** 2022-10-04

**Authors:** Eryn Wright, Gayle Pollard, Hannah Robertson, Satyamurthy Anuradha

**Affiliations:** 1 School of Public Health, The University of Queensland, Herston, QLD 4006, Australia; 2 Queensland Health, Metro South Hospital Health Service, Woolloongabba, QLD, Australia

**Keywords:** COVID-19, infectious disease control, infectious disease epidemiology, public health emerging infections, transmission

## Abstract

Household transmission plays a key role in the spread of COVID-19 through populations. In this paper, we report on the transmission of COVID-19 within households in a metropolitan area in Australia, examine the impact of various factors and highlight priority areas for future public health responses. We collected and reviewed retrospective case report data and follow-up interview responses from households with a positive case of the Delta COVID-19 variant in Queensland in 2021. The overall secondary attack rate (SAR) among household contacts was 29.6% and the mean incubation period for secondary cases was 4.3 days. SAR was higher where the index case was male (57.9% *vs.* 14.3%) or aged ≤12 years (38.7% *vs.* 17.4%) but similar for adult contacts that were double vaccinated (35.7%) and unvaccinated (33.3%). Most interview participants emphasised the importance of clear, consistent and compassionate health advice as a key priority for managing outbreaks in the home. The overall rate of household transmission was slightly higher than that reported in previous studies on the wild COVID-19 variant and secondary infections developed more rapidly. While vaccination did not appear to affect the risk of transmission to adult subjects, uptake in the sample was ultimately high.

## Introduction

Exposure to COVID-19 within households carries a significant risk of transmission given the proximity, prolonged duration and frequency of interactions that occur within the home environment. In fact, a systematic review and meta-analysis of studies on the wild COVID-19 variant determined that the pooled secondary attack rate (SAR) for households was 21.1%, indicating a far higher risk of transmission than any other exposure setting examined, including social events (SAR = 5.9%) and healthcare settings (SAR = 3.6%) [1].

Across Australia, the majority of COVID-19 cases are now being managed in home isolation. In this instance, positive cases are expected to remain at their residence unless there is an emergency, need for medical care or permission to leave has been granted by the authorities [2, 3]. Positive individuals are also instructed to limit their contact with other members of their household wherever possible to reduce further transmission [4]. Preventing transmission is particularly important in households with older adults and individuals who are immunocompromised since they remain at higher risk of severe disease, even after vaccination [5, 6]. However, restricting contact between positive cases and other household members can be challenging, especially where space within the home is limited or caregiving responsibilities are involved [7, 8].

While the household contacts of COVID-19 cases in Australia were also previously required to quarantine, they are now permitted to leave their homes in most states and territories provided that they wear a mask (those aged <12 years), are asymptomatic and have tested negative [9–14]. It is therefore likely that the introduction of home isolation and the elimination of quarantine requirements for household contacts have increased the importance of understanding household transmission to the overall management of COVID-19.

In this paper, we describe the transmission of the Delta COVID-19 variant within households in Queensland, Australia and consider factors that may have influenced the likelihood of transmission. We also report some of the common experiences and challenges individuals in those households faced during quarantine and isolation, and describe their perceptions towards COVID-19, vaccinations and the public health response surrounding the event in question. The findings will help to inform future public health efforts aimed at managing the spread of COVID-19 and other communicable diseases.

## Methods

### Background

For most of 2020 and 2021, the state of Queensland maintained a zero-community transmission approach to managing the COVID-19 pandemic within its borders. To support this strategy, strict isolation, quarantine and testing policies were introduced. One such measure that was implemented between July 2020 and August 2021 was moving all positive cases identified in the community into hospital until they were cleared of the virus. However, a change in policy allowing community-acquired cases of COVID-19 to isolate at home unless otherwise advised was introduced in August 2021, and expanded to include interstate and overseas-acquired cases in December 2021.

Cases of the SARS-CoV-2 Delta (B.1.617.2) variant of COVID-19 began to emerge in southeast Queensland communities in the second half of 2021. These comprised of part of the first cohort of COVID-19 cases in Queensland to have been managed outside of a hospital in 13 months and the very first cases of the Delta variant in the state to have isolated in household settings for any length of time.

At this time, the Queensland Government directives for home isolation stipulated that a positive individual must remain at their home and have zero contact with members of the public outside of their household (with the exception of emergencies or need for medical care) until they were cleared of the infection, i.e. 14 days had passed since the onset of symptoms and remained symptom-free for the last 72 h. They were also advised, but not required, to limit contact with uninfected members of their household wherever possible. The directives for home quarantine similarly stipulated that the contacts of a positive case must remain at home until a full incubation period of 14 days had passed since they last had contact with a positive case. The testing regime for household contacts of cases at this time comprised of three polymerase chain reaction (PCR) tests on days 3, 6 or 7 and 12 (where day 0 was the day last contact with the case). Contacts were additionally tested if they became symptomatic. In the event that a contact tested positive, the three-test regime and 14-day quarantine period then re-started for all remaining uninfected household contacts.

### Data sources

We conducted a retrospective review of the case report data for 72 people from 17 households in southeast Queensland that had at least one confirmed case of COVID-19 in 2021. Data found in the case reports was collected by the Metro South Public Health Unit in accordance with public health requirements for the management of a notifiable condition. All index cases were confirmed to be the Delta variant, and all were at home with ≥1 other people for at least part of their infection. The data extracted for positive cases included: age, sex, COVID-19 vaccination status, date of vaccinations, presence of symptoms, date of symptom onset, date of first positive PCR swab, first and last day in isolation, days admitted to hospital, days admitted to intensive care, days admitted to the hospital in the home (HiTH) programme and date cleared of infection. The data collected from household contacts who did not test positive for COVID-19 included: age, sex, days exposed to household case and days in quarantine.

An adult member of each household (≥18 years of age) was contacted over the telephone for a follow-up interview between January and February 2022. Where the first attempt at contact was unsuccessful, two further attempts were made. Potential participants were informed of the aims, nature and scope of the project, approximate duration of the interview and the fact that participation in the interview process was entirely voluntary. Interviews were semi-structured, and all were conducted by the same member of the research team using a purpose-designed list of guiding questions and prompts (see Supplementary material S1) aimed at collecting information not available in the case reports.

### Data analysis

Descriptive analyses were undertaken for all quantitative data obtained from the case reports and interviews, and SARs were calculated where appropriate. Qualitative data from interview responses were analysed thematically to identify important patterns in the perspectives, concerns and experiences of participants. Qualitative analysis was undertaken by EW and reviewed by SA and GP.

## Results

### Sample characteristics

**Fig. 1.**
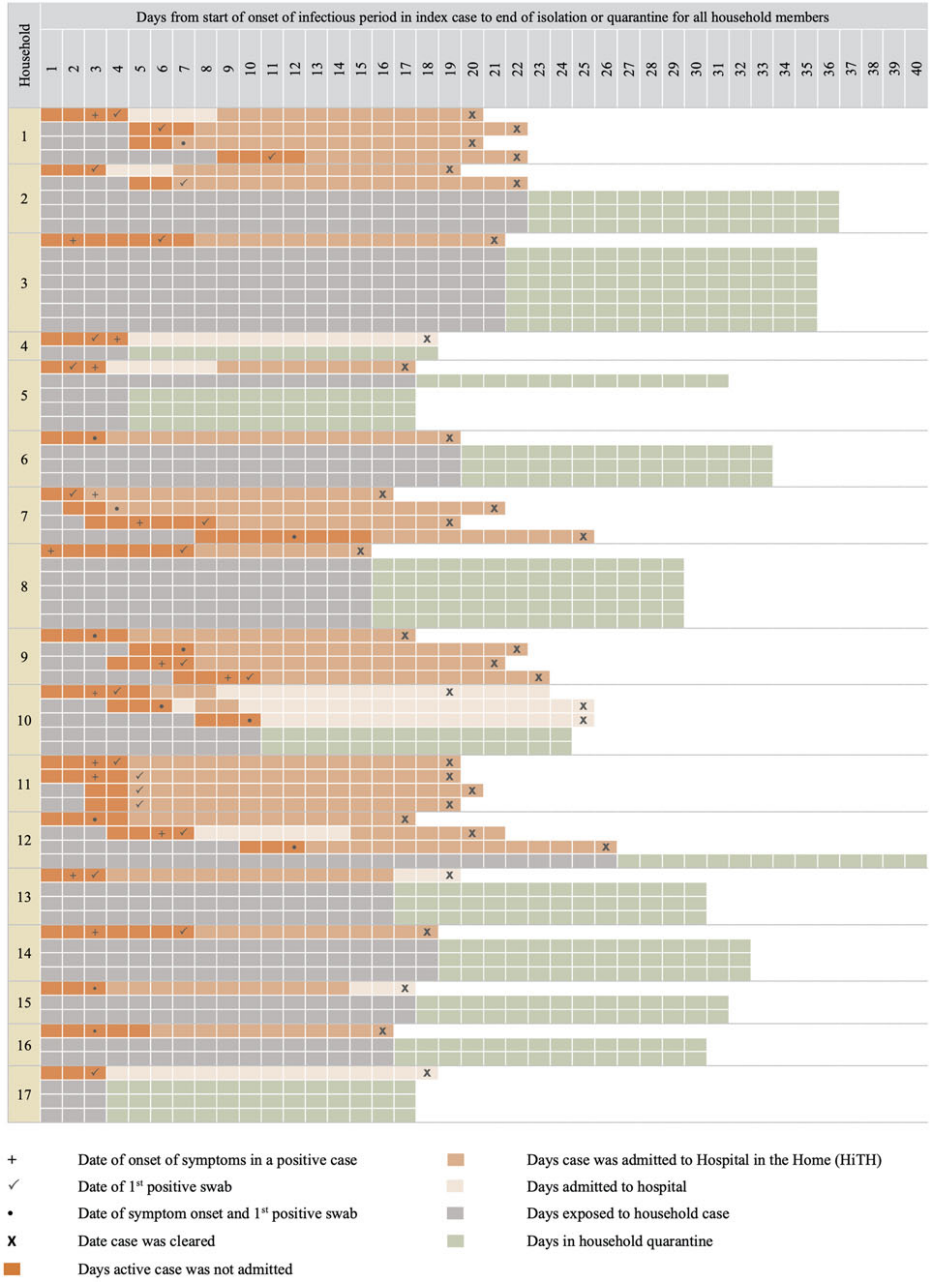
Timeline of index case onset and household exposure.

A total of 34 cases of COVID-19 were confirmed in the 17 households included for analysis, 18 of which were index cases and 16 of which were considered secondary cases. Index cases were all epidemiologically and genomically linked to known COVID-19 cases outside of the household. Two cases, each from a different household, could not be definitively excluded as potential incidents of co-infection with the index case. However, we decided to include their households as both cases had tested negative upon entering household quarantine and in both instances transmission within the household extended beyond these individuals. Thirty-eight household contacts remained uninfected at the end of their quarantine period (Fig. 1).

Key characteristics of the study sample are provided in Table 1. Fourteen (40.0%) adults in the sample were double vaccinated when the index case was diagnosed in their household, 6 (17.1%) had received one vaccine and 15 (42.9%) were unvaccinated. At the time of analysis, 29 (82.9%) adults in the sample were double vaccinated, two (5.7%) had received one vaccine and four (11.4%) remained unvaccinated or had a vaccination status that could not be confirmed. All of those who remained unvaccinated or had received only one dose had been infected during the period in question.

**Table 1.** General characteristics of the sample

Sample characteristics	Index cases	Secondary cases	All cases	Uninfected contacts	Total sample
Persons, *n*	18	16	34	38	72
Mean age, years (range)	16.0 (4–63)	27.9 (8–48)	21.6 (4–63)	29.2 (5–63)	25.6 (4–63)
Males, *n* (%)	7 (38.9)	7 (43.8)	14 (41.2)	20 (52.6)	34 (47.2)
Females, *n* (%)	11 (61.1)	9 (56.3)	20 (58.8)	18 (47.4)	38 (52.8)
Asymptomatic cases, *n* (%)	2 (11.1)	1 (6.3)	3 (8.8)	–	–
Double-vaccinated adults (≥18 years), *n* (%)	–	5 (31.3)	5 (14.7)	9 (23.7)	14 (40.0)
Adults with one vaccine dose, *n* (%)	1 (5.6)	0 (0.0)	1 (2.9)	5 (13.2)	6 (17.1)
Unvaccinated adults, *n* (%)	1 (5.6)	5 (31.3)	6 (17.6)	9 (23.7)	15 (42.9)
Mean days exposed to 1 or more cases in household (incl. HiTH)	–	4.3	–	15.0	–

The mean incubation period for secondary cases was 4.3 days (range = 1–9 days). Ten (29.4%) cases were hospitalised, none of which were admitted to the intensive care unit.

### Case report findings

Transmission occurred in seven (41.2%) of the 17 households included in the study and the characteristics of households with and without transmission based on data from the case reports are contrasted in Table 2.

**Table 2.** Characteristics of households with and without secondary transmission

Variables	Household transmission	No household transmission
Case report findings (*n* = 17 households)		
Index case aged ≤12 years, *n* (%)	5 (62.5)	3 (37.5)
Index case aged >12 years, *n* (%)	2 (22.2)	7 (77.8)
Index case male, *n* (%)	4 (66.7)	2 (33.3)
Index case female, *n* (%)	3 (27.3)	8 (72.7)
Total households, *n* (%)	7 (41.2)	10 (58.8)
Interview findings (*n* = 11 households)		
Mean number of bedrooms in household	3.0	3.6
Mean number of bedrooms per person	0.73	0.95
Mean number of bathrooms in household	2.3	2.4
Mean number of bathrooms per person	0.56	0.64
Person(s) shared a bedroom with index case, *n* (%)	3 (75.0)	1 (25.0)
No person(s) shared a bedroom with index case, *n* (%)	3 (42.9)	4 (57.1)
Index case was completely or mostly isolated from all uninfected members of the household, *n* (%)^a^	3 (60.0)	2 (40.0)
Masks were used in the household, *n* (%)	3 (37.5)	5 (62.5)
Additional cleaning was undertaken during isolation, *n* (%)	4 (50.0)	4 (50.0)
Total households, *n* (%)	6 (54.5)	5 (45.5)

a Includes households where the index case spent most of their time alone or with other infected household members and excludes households where the index case isolated with an uninfected caregiver. The most common type of mask used was surgical, followed by cloth and *n* = 95.

The SAR for all household contacts was 29.6%, and transmission was notably higher where the index case was male (SAR = 57.9% *vs.* 14.3%), aged ≤12 years (SAR = 38.7% *vs.* 17.4%) or symptomatic (SAR = 31.9% *vs.* 14.3%) (see Table 3). SAR remained higher for male index cases regardless of whether they were aged ≤12 or >12 years and for index cases aged ≤12 years regardless of their sex. Being double vaccinated did not appear to reduce SAR among adult contacts.

**Table 3.** Exposure variables and SAR among household contacts (*n* = 54) using data from case reports

Person characteristics	Infected	Exposed	SAR (%)
Index case			
Aged ≤12 years	12	31	38.7
Aged >12 years	4	23	17.4
Female	5	35	14.3
Male	11	19	57.9
Asymptomatic	1	7	14.3
Symptomatic	15	47	31.9
Household contacts			
Aged ≤12 years	5	15	33.3
Aged >12 years	11	39	28.2
Female	9	27	33.3
Male	7	27	25.9
Double-vaccinated adults	5	14	35.7
Adults with one vaccine dose	0	6	0.0
Unvaccinated adults	5	15	33.3
Exposure to household case(s) <5 days	11	19	57.9
Exposure to household case(s) ≥5 days	5	35	14.3
Total	16	54	29.6

### Interview findings

Adults from 11 (64.7%) of the 17 households were interviewed. Transmission occurred in just over half (54.5%) of these households (see Table 2) and the SAR among household contacts was 38.9%.

Analysis of the interview data showed that the majority (75%) of households in which the index case had shared a bedroom with one or more people saw further transmission of COVID-19. Most households implemented strategies aimed at reducing further transmission once the index case tested positive, including the isolation of cases and/or mask wearing. Isolation practices varied; however, transmission was lower where the index case was described as having been (at least) mostly isolated from uninfected members of the household, either alone or with a designated caregiver (SAR = 34.6% *vs.* 50.0%) (see Table 4). Mask use also appeared to decrease the risk of transmission (SAR = 29.6% *vs.* 66.7%).

**Table 4.** Exposure variables and SAR among household contacts (*n* = 35) using data from interviews

Preventative measures and exposure details	Infected	Exposed	SAR (%)
Isolation of index case			
Index case was completely or mostly isolated^a^	9	26	34.6
Index case not isolated	5	10	50.0
Person(s) shared bedroom with index case	7	16	43.8
No person(s) shared a bedroom with index case	7	20	35.0
Masks used during isolation/quarantine	8	27	29.6
By index case	5	18	27.8
By household contacts	8	22	36.4
No mask use	6	9	66.7
Dwelling characteristics			
Household type			
Single level	7	14	50.0
Two levels	7	22	31.8
House size^b^			
Small size	8	12	66.7
Medium size	6	12	50.0
Large size	0	11	0.0
Total	14	36	38.9

a Includes: index cases that spent most or all of their time alone or with other infected members of the household and index cases that spent most or all of their time with a single (uninfected) caregiver in a household with multiple people.

b Missing data from one household.

### Participant views and experiences

Ten (90.9%) of the 11 interview participants described the isolation/quarantine experience in their household as being at least somewhat challenging (or as having had specific challenges), with half of those describing it as either very challenging or more challenging than expected. Responses suggest that the process was particularly difficult for individuals in isolation/quarantine for extended periods and those for whom circumstances changed rapidly or because of factors outside of their control.

The three most common challenges described by participants were: (1) vague or inconsistent information about isolation/quarantine protocols from public health officials; (2) the detrimental impact of isolation/quarantine on mental health and wellbeing and (3) caring for children (e.g. managing emotional needs and home schooling) during the isolation/quarantine period, particularly when infection status was discordant between parent(s) and child(ren).

Specific aspects of the household isolation and quarantine compliance programme also proved difficult for some participants. For instance, respondents found it impractical to have only one point of contact in the household and a relatively short timeframe (10 min) to reply to check-in phone calls.

When asked what would have made the process easier, most participants said that they would have appreciated clearer and more consistent messaging on what precautionary measures they should take within the household, what isolation/quarantine protocols they would need to follow and/or what the plan would be for their care. Some individuals also mentioned that they would have liked to have had more information about what to expect after cases were cleared of the infection, including when they should be vaccinated, how to obtain proof of immunity and how long symptoms might linger.

While some participants felt supported by the regular check-ins and supplies offered to them, others felt that more could have been done to make the process easier such as the provision of masks and gloves. Less media attention and more compassionate interactions with compliance officers were also highlighted. A small number of those who were hospitalised stated that they would have preferred to have been given the choice to stay at home, while a small proportion of those who did stay at home felt that their situation became easier once more people in the household tested positive.

Most (72.7%) interview participants described feeling at least somewhat concerned about either getting COVID-19 or passing it on to someone else, though these feelings sometimes varied between household members. For some individuals, these feelings were eased slightly by the fact that they were vaccinated or had no underlying health conditions.

When asked about their views regarding vaccination against COVID-19, the majority (72.7%) of participants felt it was beneficial. All remaining participants stated that while they were unsure about the protection offered, they had decided to get the vaccine anyway, often because they felt it was the right thing to do. Only a small number of people expressed concerns regarding vaccine side effects, either because they themselves had experienced side effects or they had heard alarming stories. Nevertheless, most of these individuals were either still willing to get a third vaccination if advised or had already done so.

The majority of people interviewed thought that other members of the household would eventually test positive despite efforts to prevent transmission. This was often because of the level of contact that had already taken place between household members. Furthermore, complete isolation of the index case was often described as not feasible where the individual was a young child.

Views about COVID-19 going forward were mixed. While some individuals remained concerned about becoming infected or re-infected, others described feeling relatively protected through vaccine- or infection-induced immunity, especially given the reports of milder disease and better treatments.

## Discussion

In this study, we examined the transmission of the Delta COVID-19 variant within Queensland households. The overall SAR in this study (29.6%) was higher than the pooled SAR previously reported for the household transmission of the wild COVID-19 variant (21.1%) [1], further corroborating the now well-established fact that the Delta variant is more transmissible [15]. Interestingly, breakthrough infections were more common than expected given the prior evidence that double vaccination appeared to afford at least a modest level of protection against the Delta variant [16], and the fact that cases had been vaccinated only 3 weeks to 4 months before.

The average incubation period in this study (4.3 days) is the same as that reported elsewhere for the Delta COVID-19 variant and shorter than that reported for pre-Omicron non-Delta variants (5.0 days) [17]. Unlike studies on the wild COVID-19 variant that showed that SAR was highest for people exposed to household cases for ≥5 days, our analysis showed that transmission was far more likely in the first 4 days of exposure. These results are supported by research indicating that the Delta strain of the virus replicates faster within a host and is more transmissible earlier in the infection than the wild COVID-19 variant [18]. This may help to explain why some of the prevention efforts employed within households were not more effective at reducing SAR since transmission may have already occurred.

The higher rate of household transmission for index cases aged ≤12 years is supported by the findings of another study from the United States [19]. Interview participants reported that caring for younger children who were infected was quite challenging and attempts at isolating or limiting contact with these family members were often either abandoned or never undertaken to preserve their mental health and wellbeing. This could help to explain the greater likelihood of transmission from younger cases. Further examination of the interview data suggests that the higher SAR reported for households with male index cases could possibly be associated, at least in part, with smaller average house size among this group in our study. It is also worth noting that larger cohort studies comparing transmission from male and female cases have reported conflicting results [20, 21].

The data collected from household interviews add to the evidence supporting mask wearing, particularly by infected individuals, otherwise referred to as ‘source control’ [22–24]. The lower SAR in households with an asymptomatic index case also supports the results of other current research [20, 25–28]; however, these findings were significantly limited by our small sample size.

Our study showed an inverse relationship between dwelling size and the risk of COVID-19 transmission among households that agreed to be interviewed. These results emphasise the role of proximity in the spread of COVID-19 and support the use of density limits to reduce transmission.

In addition to describing the household transmission of COVID-19, our study has highlighted key aspects of the public health response. Consistent, informative and compassionate communication from health authorities was by far the most important part of the response for interview participants. Individuals often wanted or appreciated having a clear plan for the care of those in their households and well-defined pathway out of quarantine and isolation; however, the relatively sudden transition to household isolation and quarantine meant that this was not always provided. There were also negative impacts on mental health where individuals were asked to quarantine for particularly long periods and in instances where people felt they had little to no control over their isolation. A review of the literature on outbreaks that occurred prior to COVID-19 yielded similar findings, highlighting the value that these learnings hold for improving public health responses more broadly [29]. Based on participant feedback, future approaches to household quarantine and isolation should consider prioritising the following: better interagency planning and communication to improve preparedness as circumstances change and prevent conflicting messaging, flexible arrangements for communicating with households (e.g. more than one designated contact), readily accessible guidelines and updated advice on infection prevention, follow-up care and vaccination sent to those affected and efforts to limit the duration of quarantine/isolation wherever possible.

Overall, vaccination was well-perceived by most participants and all of those interviewed were willing to receive a third vaccination if advised. This is reflective of the high vaccine uptake across Queensland and Australia more broadly [30]. However, it is important to note that conflicting information about when to receive the vaccine after recovering from infection was not an uncommon problem. While a small number of interviewees remained concerned about contracting COVID-19, others felt somewhat relieved following their experience as it eliminated the sense of the unknown. Many also described feeling protected by vaccination, prior infection and/or reports of more mild disease, which may have important implications for health-related behaviours going forward.

This study has two key strengths. First, given the relatively low number of COVID-19 cases in Queensland at the time, the public health unit was able to closely follow the index cases and their households from the time the first infections were detected. This enabled the unit to collect important epidemiological data on the individuals within the household at the time of the infection and to quickly identify secondary cases among household contacts. Second, the study captures data from a critical point in the COVID-19 pandemic in Queensland, with the introduction of the Delta variant and the shift to home isolation. Policy changes have since seen the removal of nearly all other COVID-19 restrictions in Queensland, leaving the household isolation of cases as the primary means of transmission control at the population level. The information acquired through this study helps to further our understanding of COVID-19 transmission within households as public health guidelines continue the transition to living with COVID.

This study also has important limitations, one of which is the fact that PCR tests are not 100% accurate at diagnosing COVID-19 infections [31, 32]. Therefore, there is a small possibility that infections among household contacts were missed, which would compromise the accuracy of our findings. It is imperative to recognise that we were unable to discount the possibility that index cases might have been identified at different points in their infectious periods or had significantly different viral loads. We were also unable to fully account for the impacts that hospitalisation may have had on transmission where this occurred.

Interview data may have been subject to self-selection and social desirability bias given that participation was voluntary. It is particularly noteworthy that the SAR was considerably higher among households that participated in the interviews (38.9%) compared to non-responders (12.5%). Recall bias may have also been a factor as interviews were conducted after households were cleared of infection. Furthermore, as all households included in the study were from large metropolitan areas, they are unlikely to be representative of the broader Queensland and Australian populations. Finally, comprehensive statistical analyses could not be conducted because of the small sample size and uncontrolled nature of the study, thereby limiting the strength and generalisability of our findings. Ideally, larger cohort or case-control studies would be conducted to investigate the validity and significance of our observations, however given the widespread transmission of the Omicron variant, this now seems highly unlikely in an Australian setting.

In summary, the overall rate of household transmission in this case series was similar to that reported elsewhere for the Delta COVID-19 variant. Differences in transmission were observed based on the age, gender and symptom status of the index case, mask use and the size of the dwelling. While vaccination status did not appear to affect the rate of transmission to adult contacts, vaccine uptake was ultimately high (>85%) among adults in this sample. Interview findings show that clear, consistent and comprehensive communication from public health officials is an important facilitating factor for dealing with an outbreak in the home.

## Data Availability

Case report data are available by request through the Queensland Notifiable Conditions register. However, given the nature of this research and the fact that participants of this study did not agree for their interview data to be shared publicly, the data are not available.

## Supplementary material

For supplementary material accompanying this paper visit https://doi.org/10.1017/S0950268822001546. click here to view supplementary material
