# Supplementary material for: Household transmission of the Delta COVID-19 variant in Queensland, Australia: a case series
Source: Epidemiol Infect. 2022 Oct 4;150:e173. doi: 10.1017/S0950268822001546 (PMC9671917; doi:10.1017/S0950268822001546)
Supplement: Supplementary file 1 [file S0950268822001546sup001.docx]

**S1. Executive Interview questionnaire**

Good morning/afternoon, (*introduce self and project*).

My name is [name of interviewer] and I’m working with the [name of public health unit] on a quality improvement project for the management of Covid-19 in Queensland households. It’s the same unit that you would have been in contact with regarding the covid case(s) in your home. We’re interested in looking at the transmission of Covid-19 within households and were wondering if you would be willing to answer some questions about the experiences you and the rest of your household had with Covid-19. Given the rising COVID numbers, our aim is to obtain information on strategies that might help to reduce household transmission wherever possible going forward so that we can improve the quality of advice that we provide to people. Your participation is entirely voluntary. We will be interviewing a number of people in a similar fashion and when we analyse or publish the report, you can be assured that anonymity will be maintained, and no identifiable information will be used.

This interview covers a variety of topics but importantly, all of them relate to the period in 2021 when one or more of the members of your household was an active COVID-19 case. It should only take about half an hour to an hour, and you are free to skip any questions for any reason at all and if you need to stop we can always arrange to call back at a better time. We’ll also make sure to remove your name before we publish our findings and if you would like us to, we could provide you with a copy of the results. Would you be willing to answer our questions?

***Section 1: General household structure***

Firstly I’m going to start with some general questions about your home and the people living in it at the time of the COVID-19 infection.

1. So just to confirm, there were [insert number of people] living in your house at the time aged [….]?
2. Do you live in an apartment, unit or standalone house?
3. Is your home one level, two stories or split level?
4. How many bedrooms does the house have?
5. Was anyone sharing a bedroom with someone who tested positive?
6. And how many bathrooms are there in the house?
7. How many other household members shared a bathroom with the first case in the household?
8. Are any of the bathrooms ensuites to a bedroom?
9. How many separate toilets are there that are not in a bathroom?
10. If you were to describe your home, would consider it to be small, medium or large?

***Section 2: Air-conditioner use***

1. Do you have air-conditioning in your house? *If no, skip to question 13*
   1. Was the air-con used at all during the period when you had an active case in the house?

*If no, skip to question 13*

- 1. Is the air-con in all rooms of the house or only in some? If some, please detail which rooms
  2. Is it a ducted system or split system/s?

1. Did you use natural ventilation during the quarantine period? For example, keeping doors and windows open as much as possible or the case spending a lot of time outside the house during their infectious period?

***Section 3: Underlying health conditions***

1. At this time, did any members of your household have any underlying medical conditions? This would include things such as: asthma, diabetes, heart problems, auto-immune conditions, pregnancy or cancer treatment.
   1. Did that increase concerns about Covid-19 among that individual/those individuals?
   2. Did that increase concerns about Covid-19 among other members of the household?
   3. Did that influence the level of precautions taken in the household and if so, how?

***Section 4: Thoughts and attitudes about COVID-19 and COVID-19 transmission***

1. When the first case of COVID-19 was diagnosed in your household, what did you think about the need to isolate the case and quarantine the rest of the household members? *(Prompt with ‘did you think it would be easy or difficult, reasonable or impossible?)*
   1. How did you feel at the end of the quarantine and isolation period? Did you find the process relatively easy or challenging and why?
   2. Is there anything that would have helped to make the process easier?
2. When the first case of COVID-19 was diagnosed in your household, did you and the other members of your household expect you would be able to prevent further infections within your household or not? *(Prompt with ‘did everyone agree or were there different opinions?’)*
3. How would you describe the attitude of each of the members of your household to COVID-19 prior to having a positive case in the household? Overall, did they think it was very serious, fairly serious, not overly serious or not at all serious?
   1. Were there different opinions and if so, how did your household deal with these different views?

***Section 5: Vaccinations***

1. What did the members of your household think about getting a Covid-19 vaccination prior to the first individual in your household testing positive? (*Prompt with ‘were they concerned about vaccination, did they think it was beneficial or were they unsure?*)
   1. How did they feel about Covid-19 vaccinations after your household was cleared of Covid? *(Beneficial, not beneficial or harmful?*)
   2. How do the members of your household feel now about the recommendations to receive boosters? *(Beneficial, not beneficial or harmful?*)

***Section 6: Isolation practices and symptomology***

1. Was the first case identified in your household completely isolated away from all other members of the household for the entire period they were infectious?
2. Was this case cared for by one particular person in the household (e.g., did one person deal with all of the case’s dirty dishes, laundry and any cleaning that was done?)
3. Did this case have access to any outdoor areas during their infectious period? If so, how did they access the outdoors? Was it via the common areas of the house?
4. Did this case have access to common areas of the house at all, e.g., did they use the kitchen when other members of the household were not in the kitchen?
5. Were other individuals isolated from the rest of household if they started to show symptoms or tested positive?
6. Do you remember what time of day the people in your household started to develop symptoms? In the morning, afternoon, evening or during the night?

***Section 7: PPE usage***

1. Were masks used in the household? *If no, skip to question 27*
   1. What types of masks were used?
   2. Were they worn by the case(s) only, other household members only or both?
   3. How regularly were they worn by members of the household who were positive? *(Prompt with ‘Always, most of the time, sometimes, infrequently or never’)*.
   4. How regularly were they worn by members of the household around someone who was positive? *(Always, most of the time, sometimes, infrequently or never)*.
   5. How regularly were they worn by members of the household around people who were not known to be positive? *(Always, most of the time, sometimes, infrequently or never)*.
2. Were gloves used in the household? *If no, skip to question 28*
   1. Were they worn by the case(s) only, other household members only or both?
   2. How regularly were they worn by the case(s)? *(Prompt with ‘Always, most of the time, sometimes, infrequently or never’)*.
   3. How regularly were they worn by other members of the household? *(Always, most of the time, sometimes, infrequently or never)*.
3. Was hand sanitiser used in the household? *If no, skip to question 29*
   1. Was it used by the case only, other household members only or both?

***Section 8: Cleaning practices***

1. Was extra cleaning undertaken while there was a case in the household?
2. Was cleaning done in high touch areas (e.g., counters, door handles, fridge handles, couches, tv remotes, sink taps, toys, gaming remotes, electronic devices, kitchen appliances, etc.)
3. Was cleaning of common areas done more frequently during quarantine (e.g. lounge rooms, kitchen, bathrooms, offices)?
4. What cleaning products were used?
5. How frequently was cleaning undertaken?
6. Which member or members of the household undertook the extra cleaning?
7. If the case was isolated, was cleaning in their bedroom and bathroom undertaken during their isolation period?
8. How were the case’s dirty dishes, used items and food waste dealt with during quarantine? For example, was a dishwasher used or were disposable plates and utensils used?

***Section 9: Laundry practices***

1. During their isolation period, did the case have their bedding and towels changed and washed? *If no, skip to question 35*
   1. How often were they changed and washed?
   2. Did the case do the washing or did someone else do it for them?
2. During their isolation period, did the case have their clothing washed? *If no, skip to question 36*
   1. How often were their clothes washed?
   2. Did the case do the washing or did someone else do it for them?

***Section 10: Other interventions and risk factors***

1. Were there any other practices that your household put into place to try to minimise the chance of further COVID infections at the time and if so, what were they?
2. Were there any other factors that you think may have influenced the chances of Covid-19 transmission in your household?
